# Hepatic protein Carbonylation profiles induced by lipid accumulation and oxidative stress for investigating cellular response to non-alcoholic fatty liver disease in vitro

**DOI:** 10.1186/s12953-019-0149-9

**Published:** 2019-03-27

**Authors:** Peerut Chienwichai, Onrapak Reamtong, Usa Boonyuen, Trairak Pisitkun, Poorichaya Somparn, Prapin Tharnpoophasiam, Suwalee Worakhunpiset, Supachai Topanurak

**Affiliations:** 1 Faculty of Medicine and Public Health, HRH Princess Chulabhorn College of Medical Science, Chulabhorn Royal Academy, Bangkok, 10210 Thailand; 2 Department of Molecular Tropical Medicine and Genetics, Faculty of Tropical Medicine, Mahidol University, Bangkok, 10400 Thailand; 3 Center of Excellence of Antibody Research, Faculty of Tropical Medicine, Mahidol University, Bangkok, 10400 Thailand; 4 Center of Excellence in Systems Biology, Faculty of Medicine, Chulalongkorn University, Bangkok, 10330 Thailand; 5 Department of Social and Environmental Medicine, Faculty of Tropical Medicine, Mahidol University, Bangkok, 10400 Thailand

**Keywords:** Non-alcoholic fatty liver diseases, Non-alcoholic steatohepatitis, Fatty acids, Oxidative stress, Redox proteomics, Protein carbonylation, Proteomics

## Abstract

**Background:**

Non-alcoholic fatty liver disease (NAFLD) is caused by excessive accumulation of fat within the liver, leading to further severe conditions such as non-alcoholic steatohepatitis (NASH). Progression of healthy liver to steatosis and NASH is not yet fully understood in terms of process and response. Hepatic oxidative stress is believed to be one of the factors driving steatosis to NASH. Oxidative protein modification is the major cause of protein functional impairment in which alteration of key hepatic enzymes is likely to be a crucial factor for NAFLD biology. In the present study, we aimed to discover carbonylated protein profiles involving in NAFLD biology in vitro.

**Methods:**

Hepatocyte cell line was used to induce steatosis with fatty acids (FA) in the presence and absence of menadione (oxidative stress inducer). Two-dimensional gel electrophoresis-based proteomics and dinitrophenyl hydrazine derivatization technique were used to identify carbonylated proteins. Sequentially, in order to view changes in protein carbonylation pathway, enrichment using Funrich algorithm was performed. The selected carbonylated proteins were validated with western blot and carbonylated sites were further identified by high-resolution LC-MS/MS.

**Results:**

Proteomic results and pathway analysis revealed that carbonylated proteins are involved in NASH pathogenesis pathways in which most of them play important roles in energy metabolisms. Particularly, carbonylation level of ATP synthase subunit α (ATP5A), a key protein in cellular respiration, was reduced after FA and FA with oxidative stress treatment, whereas its expression was not altered. Carbonylated sites on this protein were identified and it was revealed that these sites are located in nucleotide binding region. Modification of these sites may, therefore, disturb ATP5A activity. As a consequence, the lower carbonylation level on ATP5A after FA treatment solely or with oxidative stress can increase ATP production.

**Conclusions:**

The reduction in carbonylated level of ATP5A might occur to generate more energy in response to pathological conditions, in our case, fat accumulation and oxidative stress in hepatocytes. This would imply the association between protein carbonylation and molecular response to development of steatosis and NASH.

**Electronic supplementary material:**

The online version of this article (10.1186/s12953-019-0149-9) contains supplementary material, which is available to authorized users.

## Background

Non-alcoholic fatty liver disease (NAFLD) is associated with the accumulation of excess fat in liver [1]. NAFLD was described as a medical condition where deposition of fat within the liver is more than 5% of hepatocytes [2–4]. Global prevalence of NAFLD is estimated to be as high as 25.24% [5]. NAFLD usually causes liver injuries and increases risk of other fatal diseases, such as liver fibrosis, cirrhosis, and hepatocellular carcinoma [2, 3]. Two stages of the disease have been proposed: i) simple steatosis or fat-accumulated liver and ii) non-alcoholic steatohepatitis (NASH) or fat-accumulated liver with inflammation [2, 3]. The development of NASH presumably results from induction of oxidative stress to steatotic liver [1, 2]. However, specific treatment of NASH is yet to be fully defined. In addition, the specific therapeutic targets remain to be discovered [4]. Oxidative stress, a condition in which pro-oxidants exceeds the level of antioxidants, can cause damage to cells and other macromolecules, including proteins [6, 7]. Oxidative modification of proteins can be caused by different mechanisms, such as sulfation and carbonylation [6, 8]. Protein carbonylation is the introduction of carbonyl group to proteins by the reaction with pro-oxidants or lipid peroxidation reaction [6, 9]. Typically, carbonylated proteins are aggregated and can also cause conformational change, resulting in alteration of enzyme activity [9–12]. However, recent findings indicated roles of carbonylation on protein biology. The alteration in carbonylation could be novel signaling pathway and responsive mechanism against various stress conditions [13–15].

Though the alteration of protein carbonylation has been widely studied in several diseases [11, 12, 15, 16], association of protein carbonylation with steatosis and NASH has not been fully studied yet. According to two-hit model in NAFLD, development of steatosis and NASH occurs from an increase in lipid accumulation followed by induction of oxidative stress. The subsequent oxidative stress not only causes cell injuries, but also contributes to oxidation of lipids and proteins, including protein carbonylation. The effects of protein oxidation on NAFLD progression was previously described but the specific impact of protein carbonylation on this disease has never been studied before [6, 9, 17–20]. In the present study, we aimed to identify carbonylated protein profiles in response to induction of lipid accumulation and oxidative stress in vitro. The findings of this study provide more insight into developmental process and cellular response to NAFLD, which can be useful for further development of therapeutic agents in future studies.

## Methods

### Reagents

HepG2 cells were purchased from ATCC (Rockville, MD, USA). Nile red, oleic acid, menadione (MND), trypsin and malondialdehyde (MDA) assay kit were obtained from Sigma-Aldrich (St. Louis, MO, USA), while palmitic, dihydroethidium and luminata forte chemiluminescence substrate were from Calbiochem (EMD Millipore, Billerica, MA, USA). 4′,6-diamidino-2-phenylindole, dihyrochloride (DAPI) and simple blue safe stain were purchased from Thermo Fisher Scientific (Waltham, MA, USA). Oxy-blot, protein oxidation kit, protein G beads and anti-ATP synthase subunit-α (ATP5A) antibody were obtained from Merck (Darnstadt, Germany). Immobiline dry strip (7 cm, pH 3–10 NL) and 2D clean-up kit were purchased from GE Healthcare (Little Chalfont, UK). Other chemicals were of analytical grade and used as received.

### Cell culture

HepG2 cells were cultured in minimum essential medium (MEM) with 10% fetal bovine serum (FBS) in a humidified 5% CO_2_ incubator at 37 °C.

### Induction and determination of lipid accumulation

Induction of lipid accumulation was performed according to previously published data [18, 19]. Briefly, HepG2 cells were cultured in media supplied with 200 μM oleic acid and 100 μM palmitic acid for 72 h. Level of lipid accumulation was evaluated by confocal microscopy and flow cytometry.

Confocal microscope was used to visualize cytoplasmic lipid droplet. HepG2 cells were cultured and treated with FA as mentioned above. Cells were fixed with 4% paraformaldehyde for 30 min at room temperature and stained with 3 μg/ml nile red, 0.1 μg/ml DAPI and 0.2% saponin in phosphate buffer saline (PBS) for 15 min in light-protected conditions. Cells were washed and analyzed with confocal microscope LSM700 (Zeiss, Germany) under original objective 20X with 488 nm laser and green channel for nile red, and 405 nm laser and blue channel for DAPI. Moreover, number of lipid-accumulated cells was quantified using nile red staining and flow cytometry. Cells were collected and fixed with 1% paraformaldehyde at 37 °C for 10 min and stained with 0.1 μg/ml nile red and 0.2% saponin in PBS for 10 min. Cells were injected into FL500MPL flow cytometer (Beckman Coulter, Pasadena, CA, USA). Ten thousand cells from the normal population zone were gated. High complexity and FL2-channel fluorescence intensity cells were counted as lipid-accumulated cells.

### Determination of cell proliferation after oxidative stress induction

Untreated and FA-treated cells were subsequently treated with increasing concentrations of oxidative stress inducer, MND. 3-(4,5-dimethylthiazole-2-yl)-2,5-diphenyltetrazolium bromide (MTT) assay was used to determine the highest concentration of MND that did not affect cell proliferation. The assay was performed based on a previously described method [21], with some modifications. In brief, HepG2 cells were seeded and treated with FA as mentioned above. Then, 0, 12.5, 25, 37.5, 50 μM of MND in culture media were added to untreated and FA treated cells and further incubated for 24 h. Media was removed and 0.5 mg/ml MTT was added and incubated for 4 h. MTT solution was discarded, followed by the addition of dimethyl sulfoxide. The absorbance at 540 nm was measured by a Sunrise plate reader (Tecan, Maennedorf, Switzerland).

### Lipid peroxidation reaction measurement

Both cell conditions were treated with 25 μM MND and markers of oxidative stress were detected. Cells from all the four conditions: no treatment, MND treatment, FA treatment, and FA/MND treatment, were subjected to lipid peroxidation reaction using MDA assay kit. Cells were collected and lysed by adding lysis buffer containing butylated hydroxytoluene. Cell lysates were centrifuged to remove debris and supernatant was transferred to a new tube. Thiobarbituric acid was added into each tube and incubated at 95 °C for 1.5 h. Thereafter, samples were cooled down to room temperature and the readings were recorded using SynergyH1 multi-mode fluorescence reader (Biotek, Vermont, USA) with 532 nm excitation wavelength and 553 nm emission wavelength.

### Superoxide production determination

Level of superoxide was measured for all 4 conditions utilizing flow cytometry. Cells were stained with 5 μM dihydroethidium in PBS for 30 min at 37 °C in light-protected conditions. Cells were collected and fixed with 1% paraformaldehyde. Then, cells were injected into FL500MPL flow cytometer. Ten thousand cells from the normal population zone were gated. Cell with high FL1 fluorescence signal were counted as positive cells.

### Protein extraction, carbonylation derivatization, and carbonylation detection

Proteins were extracted using lysis buffer (5 M urea, 2 M thiourea, 4% 3-((3-cholamidopropyl) dimethylammonio)-1-propanesulfonate (CHAPS), 50 mM dithiothreitol (DTT)). Cells were incubated on ice with lysis buffer for 1 h and centrifuged to collect protein lysate. Protein concentration was measured using Bradford reagent.

Derivatization of carbonylation on the proteins was performed with Oxy-blot, protein oxidation kit. In brief, 500 μg protein was denatured with 12% sodium dodecyl sulfate (SDS) and further derivatized with DNPH solution at room temperature for 25 min. Reaction was terminated by addition of neutralizing solution, followed by western blotting of carbonylated proteins to ensure successful derivatization. Protein samples were separated on 10% SDS-polyacrylamide gel electrophoresis (PAGE) and then transferred to polyvinylidene fluoride (PVDF) membrane. Membranes were blocked and probed with anti-DNPH antibody overnight at 4 °C. Thereafter, the PVDF membranes were incubated with secondary antibody, followed by incubation with luminata forte chemiluminescence substrate and the immunoreactive bands were visualized using ImageQuant LAS 400 imager (GE Healthcare). 

### Two-dimensional electrophoresis (2DE)

After DNPH derivatization, salts and detergents were removed using 2D clean-up kit, then protein concentration of each sample was measured using Bradford reagent. 100 μg of derivatized protein was mixed with immobilized pH gradient buffer (3–10 non-linear (NL)) and 1% bromophenol blue before rehydration using immobiline dry strip (7 cm, pH 3–10 (NL)) at 25 °C overnight. Ettan IPGphor 3 isoelectric focusing unit (GE Healthcare) was utilized to separate proteins according to their isoelectric points with running conditions: 300 V/30 min (Step), 1000 V/30 min (Gradient), 5000 V/90 min (Gradient), 5000 V/35 min (Step), 100 V/hold (Step) with 50 μA current per strip. The strips were reduced with 50 mM DTT in equilibration buffer (75 mM Tris pH 8.8, 6 M urea, 30% glycerol, 70 mM SDS) for 15 min and alkylated with 125 mM iodoacetamide (IAA) in equilibration buffer for 15 min. Strips were then placed over 10% SDS-PAGE and run at 150 V.

### Protein expression and carbonylation profile analysis

Protein expression profile was studied using Coomassie staining, while protein carbonylation profile was determined using anti-DNPH. For protein expression profile, the electrophoresed gels were stained with simple blue safe stain at 4 °C for overnight. Protein gels were destained with water until the background was clear and readings were taken using Image Scanner III densitometer (GE Healthcare). For protein carbonylation profiles, the electrophoresed gels were transferred to PVDF membrane and carbonylated protein spots were detected as mentioned above. Protein spots were analyzed using ImageMaster 2D Platinum software (GE Healthcare). The spots with more than 2-fold differentially expressed or carbonylated were excised for further protein identification.

### In-gel digestion and protein identification by mass spectrometry

Coomassie dye was removed from protein spots using 25 mM ammonium bicarbonate / 50% acetonitrile and the spots were further reduced by incubating at 60 °C with 4 mM DTT in 50 mM ammonium bicarbonate for 15 min. Protein spots were alkylated with 250 mM IAA at room temperature in the dark for 30 min before dehydration using 100% acetonitrile. 1 μg of trypsin was applied to dried gels, and peptides were collected after overnight incubation. Peptide solution was dried using speed vacuum device CC-105 (Tomy digital biology, Japan) and kept at − 80 °C until analysis.

Peptide samples were dissolved in 0.1% formic acid. Spots from protein expression profile were identified using an UltiMate 3000 nano-liquid chromatography system (Thermo Fisher Scientific) coupled with a micrOTOF-Q electrospray ionization quadrupole time-of-flight mass spectrometer (Bruker Daltonics, Billerica, MA, USA). The samples were injected into the machine using 2% acetonitrile and 0.1% formic acid in water as a mobile phase A and 0.1% formic acid in acetonitrile as a mobile phase B at flow rate of 300 nL/min for 60 min. Data acquisition of the mass spectrometer was controlled by Hystar software (Bruker Daltonics). The mass spectra covered mass ranges of m/z 400–3000 and 50–1500.

Protein spots from protein carbonylation profile were identified using an UltiMate® 3000 RSLCnano system (Thermo Fisher Scientific) coupled with Q Exactive Hybrid Quadrupole-Orbitrap mass spectrometer (Thermo Fisher Scientific) through EASY-Spray nano-electrospray ion source (Thermo Fisher Scientific). The samples were loaded with 5–7% acetonitrile for 5 min, 7–45% for 60 min, 45–50% for 5 min, and 50–97% for 5 min, followed by washing at 100% at 300 nL/min flow rate for 90 min. Full MS scan was done with mass ranges of m/z 200–2000. Precursor ions with + 1 and greater than + 8 charge state were excluded. Fragmentation of precursor ions was performed using Higher-energy collisional dissociation and data acquisition was collected by Thermo Xcalibur 2.2 (Thermo Fisher Scientific).

### Data analysis

A Mascot generic file (.mgf) was generated and data were searched against the Swiss-Prot database (accessed on July 1st, 2016) using Mascot version 2.4.1 (Matrix Science, London, UK). *Homo sapiens* taxonomy was selected for the search setting and one missed cleavage was allowed. The peptide tolerance was set to 200 ppm and the tandem mass spectrometry tolerance was set to 0.6 Da. Methionine oxidation (+ 16 Da), cysteine carbamidomethylation (+ 57 Da), lysine carbonylation (+ 179 Da), arginine carbonylation (+ 137 Da), threonine carbonylation (+ 178 Da), and proline carbonylation (+ 194 Da) were selected for variable modifications. Differentially expressed and carbonylated proteins were enriched with Funrich standalone algorithm [22]. The uniprot accession number and log_2_ ratio of differentially expressed and carbonylated proteins were uploaded to Funrich version 3.1.3. Pathway analyses based on biological process were performed. Differentially expressed and carbonylated proteins were further analyzed relying on Human taxonomy (ID: 9606). The analysis was performed by gene enrichment option in compare quantity mode and mapped with NAFLD relevant pathways in Reactome database.

### Immunoprecipitation and western blot analysis

Protein samples were precipitated and dissolved in immunoprecipitation buffer, 50 mM Tris, 150 mM NaCl, 1% Triton-X. 30 μg of precipitated protein was mixed and incubated with protein G beads and anti-ATP5A antibody for overnight at 4 °C, followed by beads washing with immunoprecipitation buffer solution and addition of 12% SDS into each sample. DNPH solution was added, incubated for 25 min and the reaction was stopped by addition of neutralizing solution. Samples were loaded onto 10% SDS-PAGE gel and western bot analysis was performed using anti-ATP5A and anti-DNPH.

## Results

### FA treatment promoted lipid accumulation in HepG2 cells

**Fig. 1:**
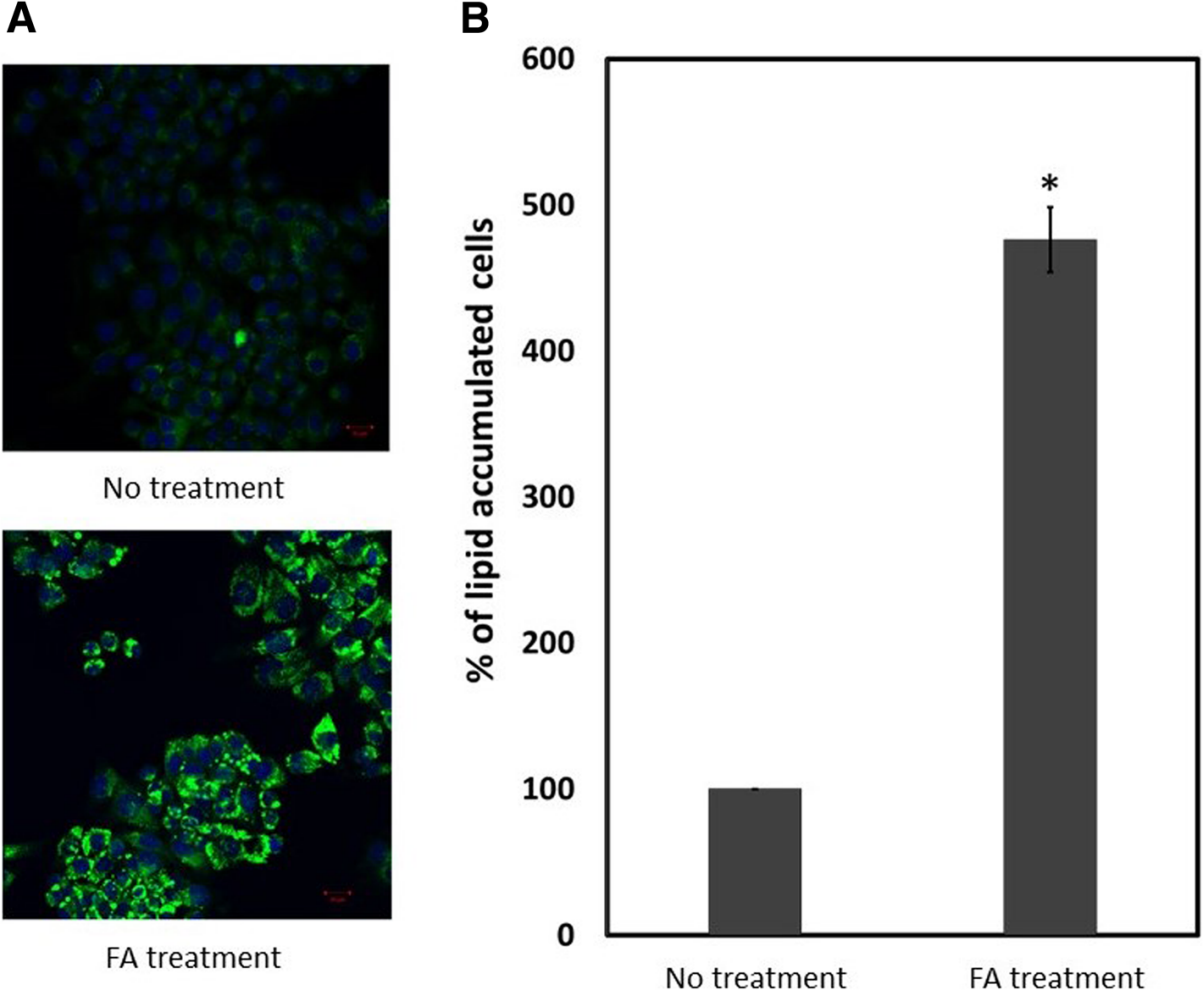
Lipid accumulation in FA treatment and no treatment of HepG2 cells. Cytoplasmic lipid droplets were observed after FA treatment. Green color represents lipid droplets and blue color represents nucleus of HepG2 cells (**a**). FA treatment increased lipid accumulated cells to 475% (**b**). The asterisk indicates statistical difference

Intracellular lipid droplets were visualized using lipid-specific fluorescence dye and confocal microscopy. Cytoplasmic lipid droplets in HepG2 cells were remarkably increased after FA treatment as shown by high signal of lipid-specific fluorescence dye (Fig. 1a). Number of lipid-accumulated cells was counted by flow cytometer and fat-accumulated cells were remarkably increased (475%) after FA treatment (Fig. 1b). Hence, this suggested that FA treatment induced lipid-accumulation in hepatocytes and this condition was used as in vitro steatosis in further experiments.

### Protein carbonylation was diminished in the presence of palmitic and oleic acid

**Fig. 2:**
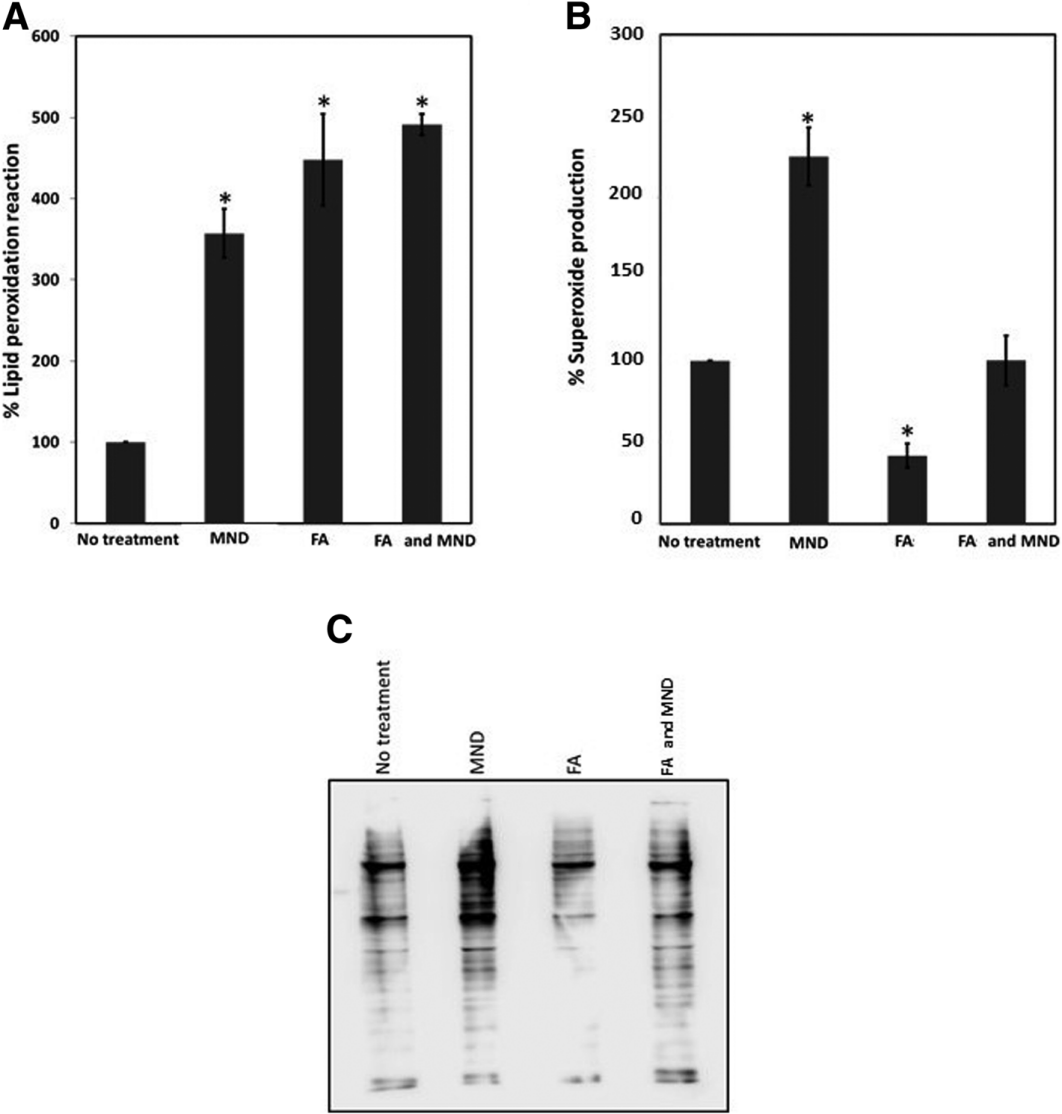
Measurement of lipid peroxidation, superoxide production and overall protein carbonylation in HepG2 with MND and FA treatment. Measurement of MDA lipid peroxidation in MND and FA treatment (**a**). Measurement of superoxide production in MND and FA treatment (**b**). Western blot analysis of DNPH derivatized proteins detecting total protein carbonylation in MND and FA treatment (**c**). The asterisks represent statistical significance (*p*-value < 0.01) comparing to no treatment

According to two-hit model, in the second stage of NAFLD, lipid-accumulated hepatocytes are exposed to oxidative stress. FA-treated HepG2 cells were subsequently treated with MND, an oxidative stress inducer. The maximum MND concentration that cells still survive is 25 μM (Additional file 1: Figure S1). Thereafter, oxidative stress markers were monitored in HepG2 with no, FA, and FA/MND treatment. Lipid peroxidation was significantly increased in both FA and FA/MND treatment (447.9 and 491.4%, respectively) (Fig. 2a). Exposure of HepG2 to FA decreased the production of superoxide (41.6%). However, when the cells were then exposed to MND, the production of superoxide was then rose up to the same level as untreated cells. (Fig. 2b). Changes in protein carbonyl formation were in good agreement with superoxide production (Fig. 2b and c). This demonstrated that we were able to induce oxidative stress to fat-accumulated cells by addition of MND.

### Protein function and enrichment

**Fig. 3:**
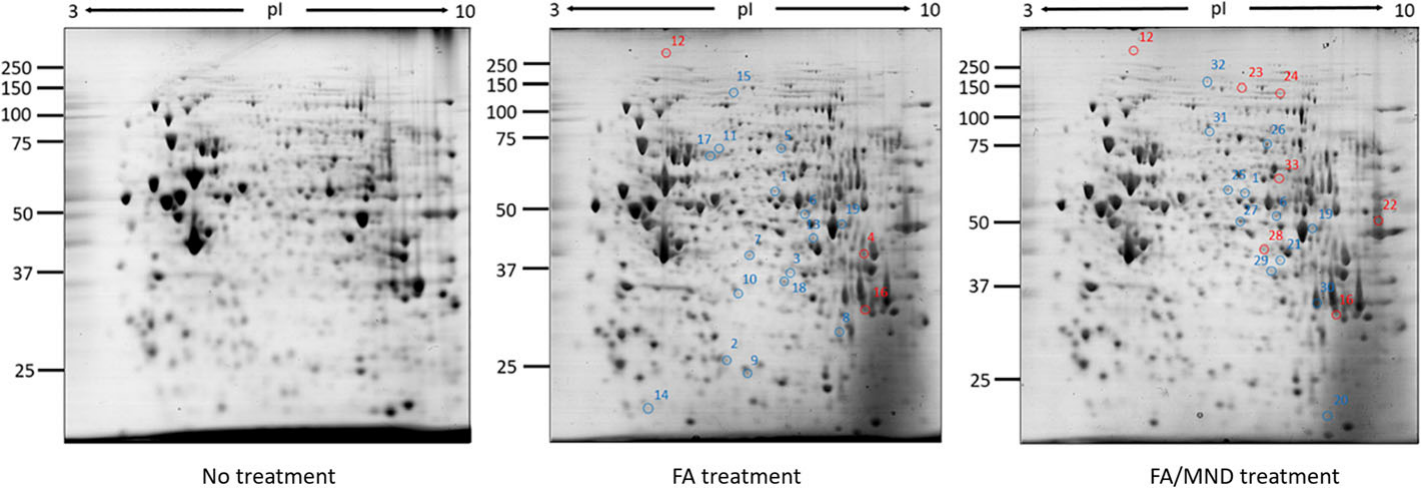
2DE images of protein expression from no treatment, FA and FA/MND treatment of HepG2 cells. Up-regulated and down-regulated protein spots were circled with blue and red color, respectively

**Fig. 4:**
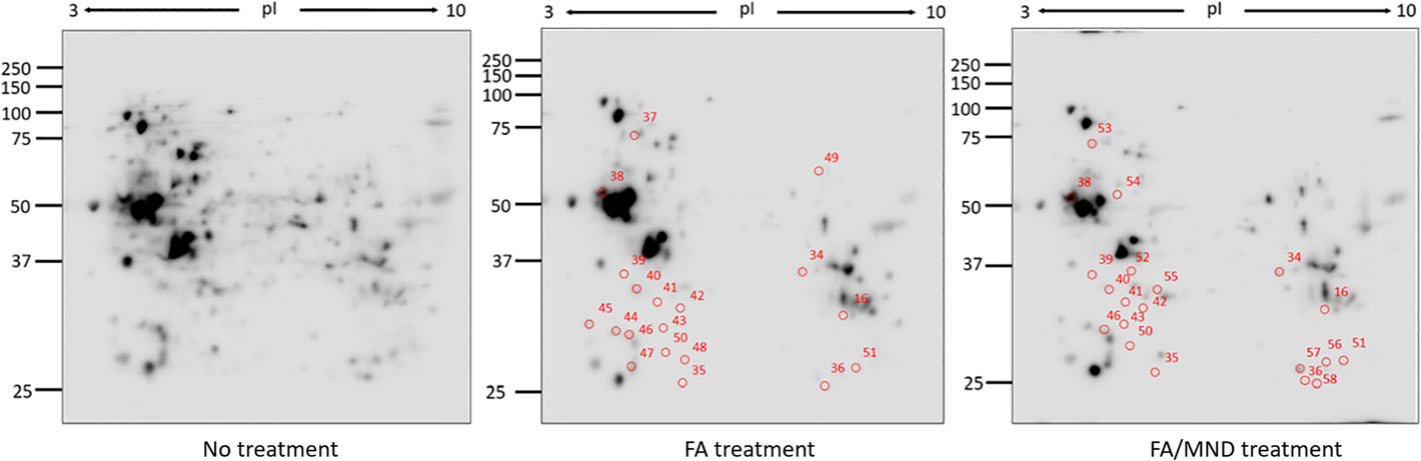
2DE images of protein carbonylation from no treatment, FA and FA/MND treatment of HepG2 cells. Carbonylated proteins exhibiting a reduction in carbonylation level were circled in red

**Fig. 5:**
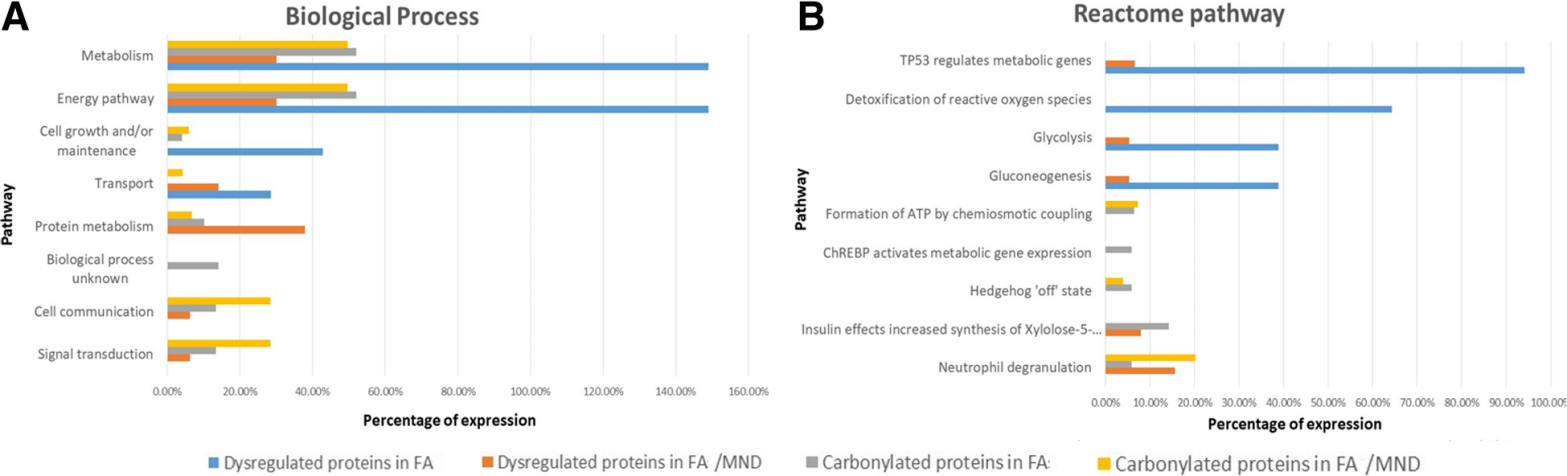
Functional classification of protein expression and carbonylation profiles. Functional classification of protein expression and carbonylation profile of FA and FA/MND treatment according to biological process (**a**) and reactome pathway (**b**) revealed the majority of identified pathways involving in energy metabolism

Protein expression and carbonylation profiles were investigated for untreated, FA-treated and FA/MND-treated cells using 2DE proteomics. Expression of 19 proteins and carbonylation of other 19 proteins were altered after FA treatment (Figs. 3 and 4, Tables 1 and 3). Interestingly, Stitch pathway analysis of differentially expressed proteins was found to correlated with NAFLD pathway (KEGG pathway ID: 04932, false discovery rate: 5.73e-08), indicating that induction of lipid accumulation by FA treatment in this study could be used for investigation of in vitro steatosis. For FA/MND treatment, 19 proteins were differentially expressed and 20 proteins showed alteration in carbonylation level (Fig. 3 and 4 and Tables 2 and 4). However, no association with NAFLD was identified using Stitch pathway analysis. Importantly, using Funrich pathway analysis tool, most of differentially expressed and carbonylated proteins from FA-treated and FA/MND-treated cells were involved in metabolic pathways (Fig. 5). In particular, the carbonylated proteins were mostly mapped in the pathways related to energy and fatty acid metabolisms, cell signaling, p53, cell cycle, inflammation, fibrosis, interleukin, insulin and apoptosis. We hypothesized that alteration in protein carbonylation in those pathways, especially in energy metabolism, by introduction of oxidative stress, may contribute to progression of simple steatosis to NASH.

**Table 1:** Protein expression profile by comparison of No treatment and FA treatment of HepG2)

Spot No.	Protein ID	Protein name	2DE		Prediction		Mascot score	Sequence coverage	Log_2_ Ratio*
			MW(kDa)	pI	MW(kDa)	pI			
1	O94925	Glutaminase kidney isoform, mitochondrial	57	6.9	73.4	7.85	88	9	1.06
2	P78417	Glutathione S-transferase omega-1	26	5.8	27.5	6.24	261	29	1.28
3	Q15365	Poly(rC)-binding protein 1	39	7.1	37.5	6.66	349	32	2.03
4	P00558	Phosphoglycerate kinase	42	8.8	44.6	8.3	216	20	−1.58
5	Q9NUB1	Acetyl-coenzyme A synthetase 2-like, mitochondrial	75	7.0	74.8	6.66	901	38	1.02
6	P28838	Cytosol aminopeptidase	49	7.5	56.1	8.03	116	9	1.22
7	P30740	Leukocyte elastase inhibitor	41	6.3	42.7	5.9	146	20	1.00
8	P21796	Voltage-dependent anion-selective channel protein 1	31	8.3	30.7	8.62	29	7	1.12
9	P30084	Enoyl-CoA hydratase, mitochondrial	25	6.3	31.3	8.34	37	21	1.06
10	P37837	Transaldolase	35	6.0	37.5	6.36	171	10	1.27
11	P11142	Heat shock cognate 71 kDa protein	77	5.6	70.8	5.37	163	16	1.50
12	Q562R1	Beta-actin-like protein 2	303	4.3	40.0	5.39	33	4	−1.15
13	O57874	Isocitrate dehydrogenase [NADP] cytoplasmic	45	7.7	46.6	6.53	1168	60	1.99
14	O00217	NADH dehydrogenase [ubiquinone] iron-sulfur protein 8, mitochondrial	22	4.2	23.7	6.00	34	4	1.31
15	O43933	Peroxisome biogenesis factor 1	128	5.9	14.2	5.91	41	2	1.00
16	P00338	L-lactate dehydrogenase A chain isoform 3	33	8.8	36.6	8.44	1732	80.1	−2.00
17	P02771	Alpha-fetoprotein	73	5.3	68.6	5.48	63	5	1.13
18	Q92890	Ubiquitin fusion degradation protein 1 homolog	37	7.1	34.5	6.27	109	20	2.38
19	P06733	Alpha-enolase	47	8.5	47.1	7.01	421	35	2.43

*Log2 Ratio refers to Log2 ratio of spot intensity from FA treatment to No treatment

**Table 2:** Protein expression profile by comparison of No treatment and FA/MND treatment of HepG2

Spot No.	Protein ID	Protein name	2DE		Prediction		Mascot score	Sequence coverage	Log_2_ Ratio*
			MW(kDa)	pI	MW(kDa)	pI			
1	O94925	Glutaminase kidney isoform, mitochondrial	57	6.9	73.4	7.85	88	9	1.22
6	P28838	Cytosol aminopeptidase	49	7.5	56.1	8.03	116	9	1.05
12	Q562R1	Beta-actin-like protein 2	303	4.3	40.0	5.39	33	4	−1.29
16	P00338	L-lactate dehydrogenase A chain isoform 3	33	8.8	36.6	8.44	1732	80.1	−1.40
19	P06733	Alpha-enolase	47	8.5	47.1	7.01	421	35	1.59
20	Q06830	Peroxiredoxin-1	22	8.4	22.1	8.27	545	55	2.63
21	P16930	Fumarylacetoacetase	41	7.6	46.3	6.46	118	6	1.01
22	P68104	Elongation factor 1-alpha 1	50	9.8	50.1	9.1	161	4	−2.64
23	P33176	Kinesin-1 heavy chain	137	6.7	109.6	6.12	136	7	−1.15
24	P43243	Matrin-3	129	7.5	94.6	5.87	228	19	− 1.65
25	Q13733	Sodium/potassium-transporting ATPase subunit alpha-4	57	6.5	114.1	6.23	28	2	1.17
26	Q12931	Heat shock protein 75 kDa, mitochondrial	79	7.3	80.1	8.3	60	10	1.06
27	P06733	Alpha-enolase	48	6.8	47.1	7.01	102	27	1.00
28	P61163	Alpha-centractin	43	7.3	42.6	6.19	92	8	−1.00
29	Q15365	Poly(rC)-binding protein 1	40	7.4	37.4	6.66	249	38	1.02
30	O60218	Aldo-keto reductase family 1 member B10	35	8.5	36.0	7.66	974	45	1.04
31	Q16891	MICOS complex subunit MIC60	85	6.1	83.6	6.08	113	87	1.52
32	P14136	Glial fibrillary acidic protein	162	6.0	49.8	5.42	93	2	2.38
33	O43933	Peroxisome biogenesis factor 1	63	7.6	142.8	5.91	39	2	−2.47

*Log2 Ratio refers to Log2 ratio of spot intensity from FA/MND treatment to No treatment)

### Carbonylation of ATP5A was decreased in FA and FA/MND treatment

Pathway analysis suggested that the majority of differentially expressed and carbonylated proteins was involved in energy pathway. Hence, in this study, we focused on proteins those play role in cellular energy generation. ATP5A was selected for further analysis because it is a key enzyme in oxidative phosphorylation (OXPHOS), which is responsible for energy production.

**Fig. 6:**
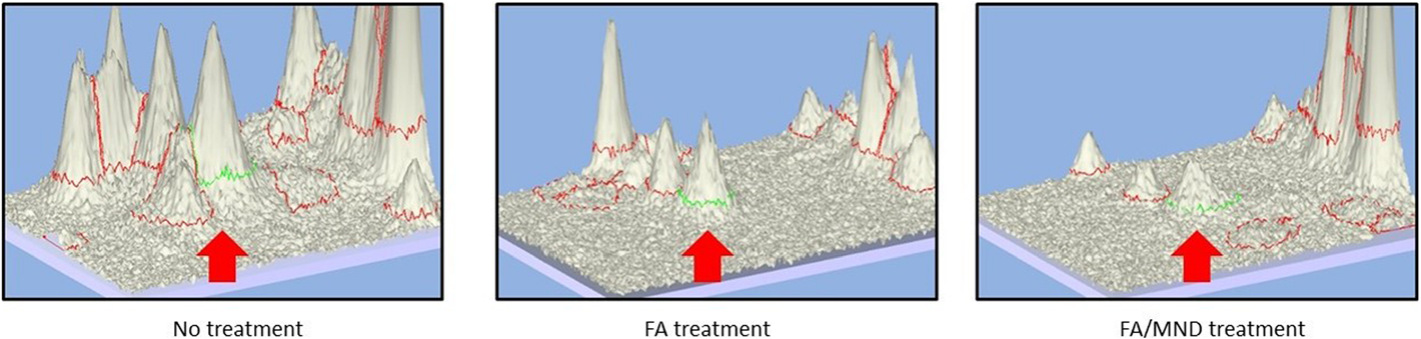
Three dimension image analysis showing carbonylated ATP5A spot in FA treatment and FA/MND treatment. Spot peaks of ATP5A are indicated by red arrow

2 DE results demonstrated that level of carbonylation of ATP5A, (Fig. 4, spot 51) was 3-fold and 3.7-fold decreased in FA and FA/MND treatment, respectively (Fig. 6, Tables 3 and 4), whereas level of ATP5A expression remained unchanged. ATP5A was further verified by anti-DNPAH antibody against derivatized carbonylation of immunoprecipitated ATP5A in which immunoblotting results were corresponding to 2DE, (Additional file 2: Figure S2).

**Table 3:** Protein carbonylation profile by comparison of No treatment and FA treatment of HepG2

Spot No.	Protein ID	Protein name	2DE		Prediction		Mascot score	Sequence coverage	Log_2_ Ratio*
			MW(kDa)	pI	MW(kDa)	pI			
16	P00338	L-lactate dehydrogenase A chain isoform 3	39	8.6	36.6	8.44	1732	80.1	0.00
34	P04057	Fructose-bisphosphate aldolase A isoform 1	48	7.7	13.2	8.52	2058	86.5	− 1.36
35	P78330	Phosphoserine phosphatase	29	5.5	25	5.51	1246	84	0.00
36	P35270	Sepiapterin reductase	28	8.7	28.0	8.2	820	67	−3.47
37	P11021	78 kDa glucose-regulated protein	86	4.3	72.3	5.0	277	12	−1.65
38	P68371	Tubulin beta-4B chain	66	3.6	49.8	4.7	2177	83.4	−1.43
39	Q9Y3F4	Serine-threonine kinase receptor-associated protein	46	4.1	38.4	4.9	56	5	−2.06
40	P55735	Protein SEC13 homolog	43	4.4	35.5	5.2	88	19	−1.03
41	Q13733	Sodium/potassium-transporting ATPase subunit alpha-4	40	4.9	114.1	6.2	29	2	0.00
42	Q15181	Inorganic pyrophosphatase	38	5.6	32.6	5.5	309	35	−2.19
43	Q9HC38	Glyoxalase domain-containing protein 4	36	5.2	34.7	5.4	31	4	0.00
44	P08758	Annexin A5	36	4.0	35.9	4.9	1419	58	−1.29
45	P12004	Proliferating cell nuclear antigen	37	3.4	28.7	4.5	406	62	−1.29
46	Q15691	Microtubule-associated protein RP/EB family member 1	35	4.3	29.9	5.0	60	27	−1.74
47	Q99943	1-acyl-sn-glycerol-3-phosphate acyltransferase alpha	30	4.4	31.7	9.48	38	3	−1.43
48	P35232	Prohibitin	31	5.6	29.8	5.5	1199	59	−1.84
49	P29401	Transketolase	74	8.5	67.8	7.5	791	41	−3.47
50	Q9UL46	Proteasome activator complex subunit 2	32	5.1	27.4	5.5	723	74	0.00
51	P25705	ATP synthase subunit alpha, mitochondrial	31	9.4	59.7	5.1	1222	42.7	−1.58

* Log2 Ratio refers to Log2 ratio of spot intensity from FA treatment to No treatment

**Table 4:** Protein carbonylation profile by comparison of No treatment and FA/MND treatment of HepG2

Spot No.	Protein ID	Protein name	2DE		Prediction		Mascot score	Sequence coverage	Log_2_ Ratio*
			MW(kDa)	pI	MW(kDa)	pI			
16	P00338	L-lactate dehydrogenase A chain isoform 3	39	8.6	36.6	8.44	1732	80.1	0.00
34	P04057	Fructose-bisphosphate aldolase A isoform 1	48	7.7	13.2	8.52	2058	86.5	−2.00
35	P78330	Phosphoserine phosphatase	29	5.5	25	5.51	1246	84	−2.40
36	P35270	Sepiapterin reductase	28	8.7	28.0	8.2	820	67	−2.64
38	P68371	Tubulin beta-4B chain	66	3.6	49.8	4.7	2177	83.4	−1.03
39	Q9Y3F4	Serine-threonine kinase receptor-associated protein	46	4.1	38.4	4.9	56	5	−1.40
40	P55735	Protein SEC13 homolog	43	4.4	35.5	5.2	88	19	0.00
41	Q13733	Sodium/potassium-transporting ATPase subunit alpha-4	40	4.9	114.1	6.2	29	2	0.00
42	Q15181	Inorganic pyrophosphatase	38	5.6	32.6	5.5	309	35	−2.64
43	Q9HC38	Glyoxalase domain-containing protein 4	36	5.2	34.7	5.4	31	4	0.00
46	Q15691	Microtubule-associated protein RP/EB family member 1	35	4.3	29.9	5.0	60	27	−2.06
50	Q9UL46	Proteasome activator complex subunit 2	32	5.1	27.4	5.5	723	74	0.00
51	P25705	ATP synthase subunit alpha, mitochondrial	31	9.4	59.7	5.1	1222	42.7	−1.89
52	A0A024QZX5	Serpin B6 isoform B	47	4.9	43.0	5.1	1876	82.4	−1.58
53	P11021	78 kDa glucose-regulated protein	85	4.1	72.3	5.0	2083	56	−1.06
54	P10809	60 kDa heat shock protein, mitochondrial	66	4.8	61.0	5.7	329	26	0.00
55	Q5T6V5	UPF0553 protein C9orf64	43	5.5	39.0	5.6	191	11	−3.64
56	P09661	U2 small nuclear ribonucleoprotein A’	33	9.0	28.4	8.7	131	4	−1.69
57	P54819	Adenylate kinase 2, mitochondrial isoform a	30	8.6	26.4	7.67	1248	40	−1.25
58	P04075	Fructose-bisphosphate aldolase A	27	8.9	39.4	8.3	47	6	−2.64

*Log2 Ratio refers to Log2 ratio of spot intensity from FA/MND treatment to No treatment

### Carbonylation was found on nucleotide-binding region of ATP5A

**Fig. 7:**
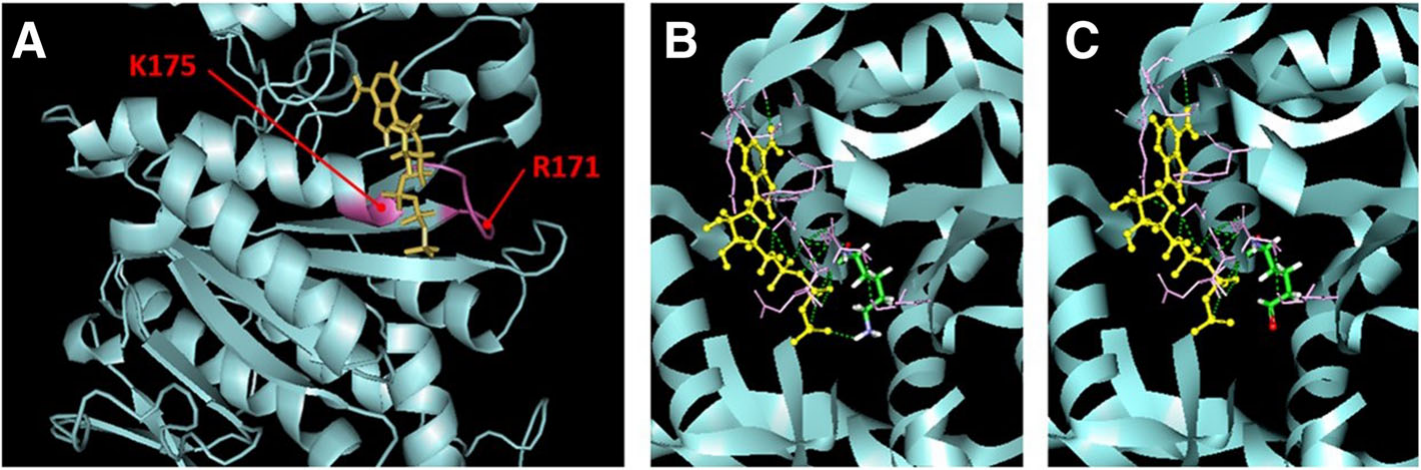
3D structure of ATP5A (PDB ID: 1NBM). Nucleotide binding region (pink), including K175 and R171 carbonylation sites, interacts with ATP (yellow) (**a**). Illustration of K175 amino side chain (green) interacts with ATP; nitrogen atom of the side chain (purple) and hydrogen atom (white) interact (green dash) with phosphate group of ATP (yellow) (**b**). Carbonylation at K175 (Red) contributes to inability of ATP binding (yellow) to nucleotide-binding region (**c**). The graphical representation was constructed using Discovery Studio Visualizer-Accelrys

To identify carbonylation sites, MS results of the carbonylated proteins were subjected to further analysis using MASCOT search engine with parameters set up against carbonylation of four amino acid residues: lysine, arginine, threonine, and proline. Seventeen carbonylation sites were identified from 6 proteins (Additional file 3: Table S1). K132, R171, K175, and R219 residues were identified as carbonylation sites of ATP5A in which R171 and K175 are located in ATP5A nucleotide-binding region (residues 169–176) [23] (Additional file 4: Figure S3, Fig. 7a). The presence of carbonylation on the nucleotide-binding region of this protein possibly affects its activity, hence, 3D structure of ATP5A (PDB: 1NBM) was visualized and the interactions between this protein and its substrate, ATP, was studied [24]. 3D structure revealed that ATP molecule interacts with 12 amino acid residues including K175, which its amino group directly interacts with phosphate group of ATP molecule (Fig. 7b). Carbonylation at amino group of K175 affected chemical property of this residue and then interrupted interaction with ATP substrate (Fig. 7c).

## Discussion

Our findings suggested that FA and FA/MND treatments altered cellular energy metabolism as well as other pathways that might be associated with steatosis and NASH. Previous in vitro studies showed that FA-treated hepatocyte cell line exhibited similar phenotypes to those observed in patients and in vivo studies [19, 25, 26]. In addition to lipid accumulation, treatment of FA also induced oxidative stress, lipid peroxidation reaction, secretion of inflammatory cytokines, and mitochondrial dysfunction in hepatocyte line, which are key events of NAFLD [19, 25, 26].

There were a number of studies described the development of NASH from steatosis through induction of oxidative stress [6, 9, 17]. Whereby, the present study attempted to simulate this event by exposing fat-accumulated HepG2 to oxidative stress inducer, MND [27], in which the results showed that MND elevated level of lipid peroxidation and superoxide production in FA-treated cells. Surprisingly, FA treatment decreased superoxide production whereas this increased level of lipid peroxidation (Fig. 2a-b). In previous studies, lipid accumulation in hepatocytes causes lipid peroxidation and mitochondrial dysfunction, leading to increased oxidative stress [17, 28]. Reduction of superoxide in fat-accumulated cells (Fig. 2b) was hypothesized as effects of FA treatment on OXPHOS. FA had been reported as inhibitor and uncoupler of OXPHOS in previous study [29], which OXPHOS is the major source of pro-oxidant production, including superoxide [30]. Additionally, decrease in activity of OXPHOS enzymes is common in both NAFLD patients and FA-treated hepatocyte line [25], therefore, superoxide production might be diminished through reduction of OXPHOS activity after FA treatment. Another possibility is that FA treatment reduced the release of pro-oxidant from mitochondria. During high-energy supply, such as high accumulation of lipid; the release of pro-oxidants from mitochondria is maintained at minimal level by increasing level of antioxidants in mitochondria [29, 31]. This led to reduction of pro-oxidants, including superoxide.

Oxidative stress induces protein post-translational modification by several mechanisms, including carbonylation [6, 8]. Currently, effects of protein carbonylation on development and progression of NAFLD are not fully understood. In this study, pathway analysis using Stitch, KEGG and Reactome databases demonstrated that majority carbonylated proteins are associated with NALFD pathways such as energy and fatty acid metabolisms, inflammation, fibrosis, P53 and interleukin. This study provided the preliminarily results which suggested that protein carbonylation might be involved in progression of simple steatosis to NASH.

Previously, carbonylation of protein was considered as an irreversible consequence of oxidative protein damage, resulting in aggregation and degradation of proteins and, finally, leading to cellular dysfunction [9, 10]. However, recent studies have described that protein carbonylation can serve as thiol-mediated signal transduction, where thioredoxin activity plays fundamental role in decreasing the level of protein carbonylation [13, 14, 32]. Carbonylation on proteins can induce conformational change and affect interaction with their partners in ligand-receptor manner [13, 14]. In this study, reduction in carbonylation level was observed for many proteins, especially proteins in energy and fatty acid metabolisms. Importantly, we observed a decrease in carbonylation level of ATP5A, a subunit of ATP synthase which is a major ATP-producer of OXPHOS. In fact, carbonylated form of this protein is commonly found in physiological conditions in which it is slightly appeared during high demand of energy condition, for instance, in muscles after exercise [33–38]. It is interesting to investigate the effects of carbonylation on ATP5A activity which could, then, play important role in energy production of the cells. We have identified the carbonylation sites on ATP5A using mass spectrometry.

Regarding MS results and protein database search against specific carbonylation at lysine, arginine, threonine and proline. Two out of four carbonylation sites of ATP5A (R171 and K175) are located in nucleotide-binding region. Previously, K175 was found to be essential for ATP5A activity since this site directly interacts with ATP molecule [23]. The positive charge of amino group of K175 interacts with negative charge of the phosphate group of newly synthesized ATP [23]. Replacement of K175 with isoleucine caused weak assemble of ATP with ATP synthase, contributing to instability of ATP-ATP synthase complex and protein malfunction [39]. Carbonylation changed the polarity of lysine side chain from positively charge (amino group) to relatively negative of aminoadipic semialdehyde. The charge switching of this residue might critically hinder the interaction with ATP and affect the activity of ATP5A.

A number of studies described energy disturbance in NAFLD patients [40, 41]. The excessive fat interrupts energy metabolism by altering the function of OXPHOS and decreasing ATP production. In animal model, OXPHOS became dysfunction and ATP production was decreased after 3 weeks of NAFLD induction. Interestingly, 7 weeks after NAFLD induction, ATP production returned to normal, indicating cellular adaptation to the disease [25, 42]. Excessive FA reduced assembly of OXPHOS units and inhibited their activities [26]. In this study, we found lower level of carbonylation of ATP5A after FA and FA/MND treatments, comparing to no treatment (Fig. 6, Additional file 2: Figure S2). The decrease in carbonylation level on ATP5A might suggest cellular mechanism in response to oxidative stress and fat accumulation. Under normal condition, it was hypothesized that hepatocytes maintain the optimum level of ATP via carbonylation of ATP5A. After FA and oxidative stress induction, OXPHOS and ATP production are decreased. As a consequence, in order to maintain ATP level, carbonylation on ATP5A is decreased. Under these conditions, the reduction in carbonylation level of ATP5A is the adaptive mechanism to enhance ATP production of oxidative phosphorylation.

## Conclusions

In summary, the present study demonstrated that protein carbonylation is likely to be involved in cellular response to development of NAFLD. Changes in level of expression and carbonylation of proteins in energy metabolism were early adaptive mechanism from lipid accumulation in oxidative stress conditions. Moreover, 3D structural model of ATP5A suggested that carbonylation sites were located in nucleotide-binding region. Carbonylation at K175 blocks the binding to ATP. It could be concluded that reduction of carbonylation of ATP5A at K175 enhances ATP production. This possibly suggested the adaptive mechanism to maintain ATP level under excessive lipid and oxidative stress conditions.

## Additional files

Additional file 1: **Figure S1.** Percentage of HepG2 cell proliferation of FA treatment in the presence of different concentrations of MND (Percentage of cell proliferation was normalized by 0 μM of MND). 25 μM was the highest concentration of MND that did not reduce proliferation of untreated and FA treated cells. The asterisks indicate statistical difference. (JPG 59 kb)

Additional file 2: **Figure S2.** Carbonylation levels of ATP5A in FA treatment and FA/MND treatment. Western blot analysis of protein expression of immunoprecipitated ATP5A probed with anti-ATP5A (A). Western blot analysis of carbonylation level of immunoprecipitated ATP5A probed with anti-DNPH (B). (JPG 590 kb)

Additional file 3: **Table S1.** Carbonylated sites identified by mass spectrometry of carbonylated proteins detected with redox proteomics. (DOCX 15 kb)

Additional file 4: **Figure S3.** MS/MS spectrum of ATP5A peptides showing carbonylation on K132 (A), R171 and K175 (B), and R219 (C) residue. Underlined alphabets refer to residues with carbonylation. (JPG 97 kb)

## Abbreviations

2DE: Two-dimensional gel electrophoresis
ACN: Acetonitrile
ATP5A: ATP synthase subunit α
CHAPS: 3-((3-cholamidopropyl)dimethylammonio)-1-propanesulfonate
DAPI: 4′,6-diamidino-2-phenylindole, dihyrochloride
DNPH: Dinitrophenyl hydrazide
DTT: dithiothreitol
FA: Fatty acids
FBS: Fetal Bovine Serum
IAA: Iodoacetamide
MDA: Malondialdehyde
MEM: Minimum Essential Medium
MND: Menadione
MS: Mass spectrometry
MTT: 3-(4,5-dimethylthiazole-2-yl)-2,5-diphenyltetrazolium bromide
NAFLD: Nonalcoholic fatty liver disease
NASH: Nonalcoholic steatohepatitis
OXPHOS: Oxidative phosphorylation
PAGE: Polyacrylamide Gel Electrophoresis
PBS: Phosphate Buffer Saline
PVDF: Polyvinylidene fluoride
SDS: Sodium Dodecylsulfate
