# Supplementary figures and images for: Hepatic protein Carbonylation profiles induced by lipid accumulation and oxidative stress for investigating cellular response to non-alcoholic fatty liver disease in vitro
Source: Proteome Sci. 2019 Mar 27;17:1. doi: 10.1186/s12953-019-0149-9 (PMC6438040; doi:10.1186/s12953-019-0149-9)

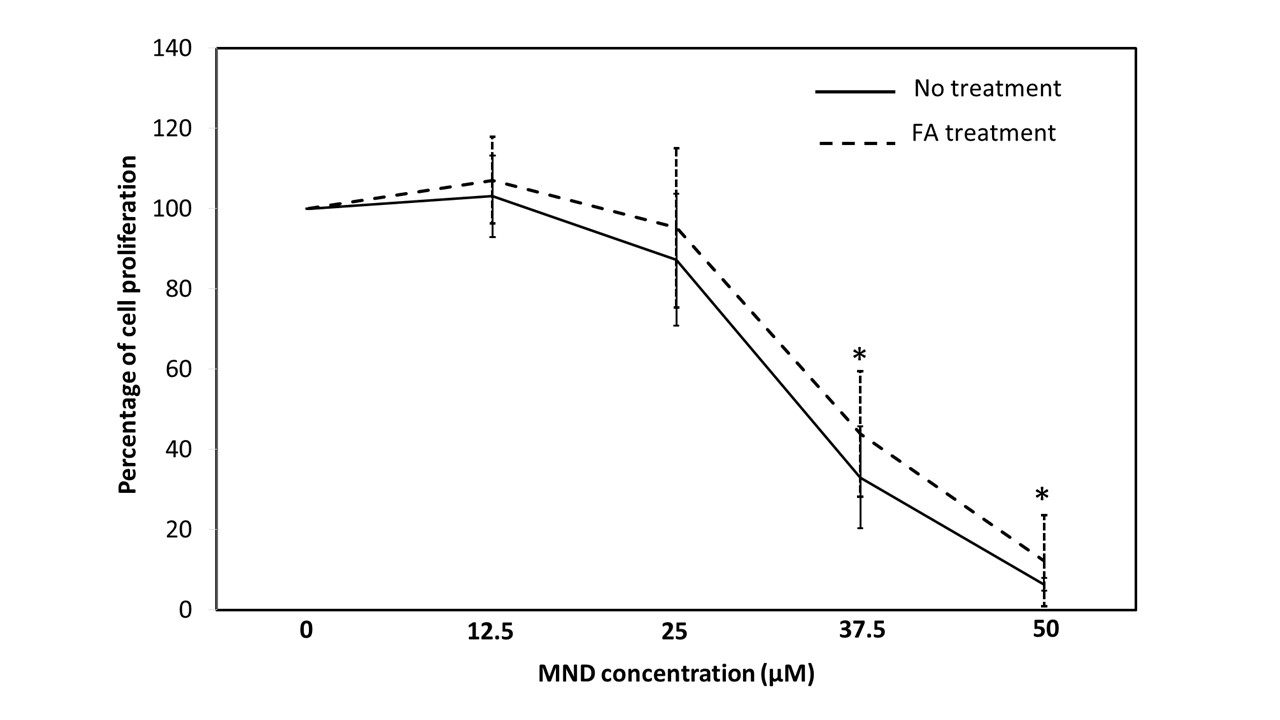

Supplement: Supplementary file 1 — Figure S1. Percentage of HepG2 cell proliferation of FA treatment in the presence of different concentrations of MND (Percentage of cell proliferation was normalized by 0 μM of MND). 25 μM was the highest concentration of MND that did not reduce proliferation of untreated and FA treated cells. The asterisks indicate statistical difference. (JPG 59 kb) [file 12953_2019_149_MOESM1_ESM.jpg]

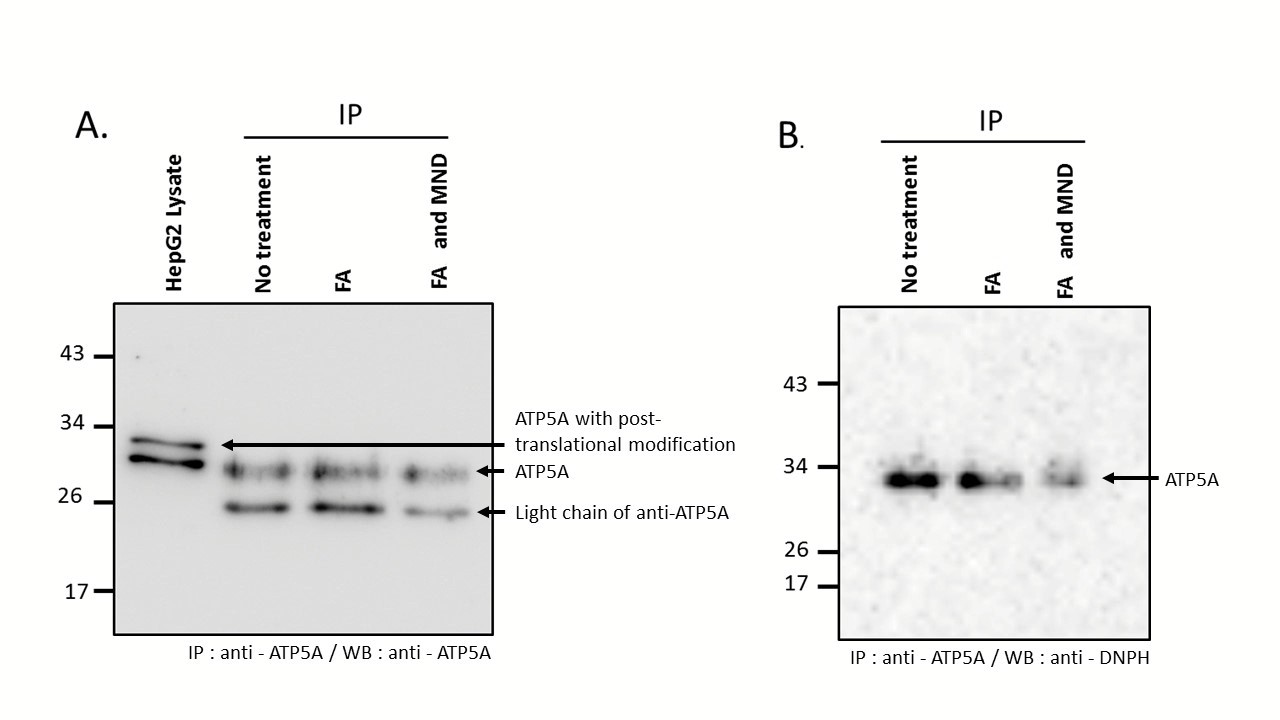

Supplement: Supplementary file 2 — Figure S2. Carbonylation levels of ATP5A in FA treatment and FA/MND treatment. Western blot analysis of protein expression of immunoprecipitated ATP5A probed with anti-ATP5A (A). Western blot analysis of carbonylation level of immunoprecipitated ATP5A probed with anti-DNPH (B). (JPG 590 kb) [file 12953_2019_149_MOESM2_ESM.jpg]

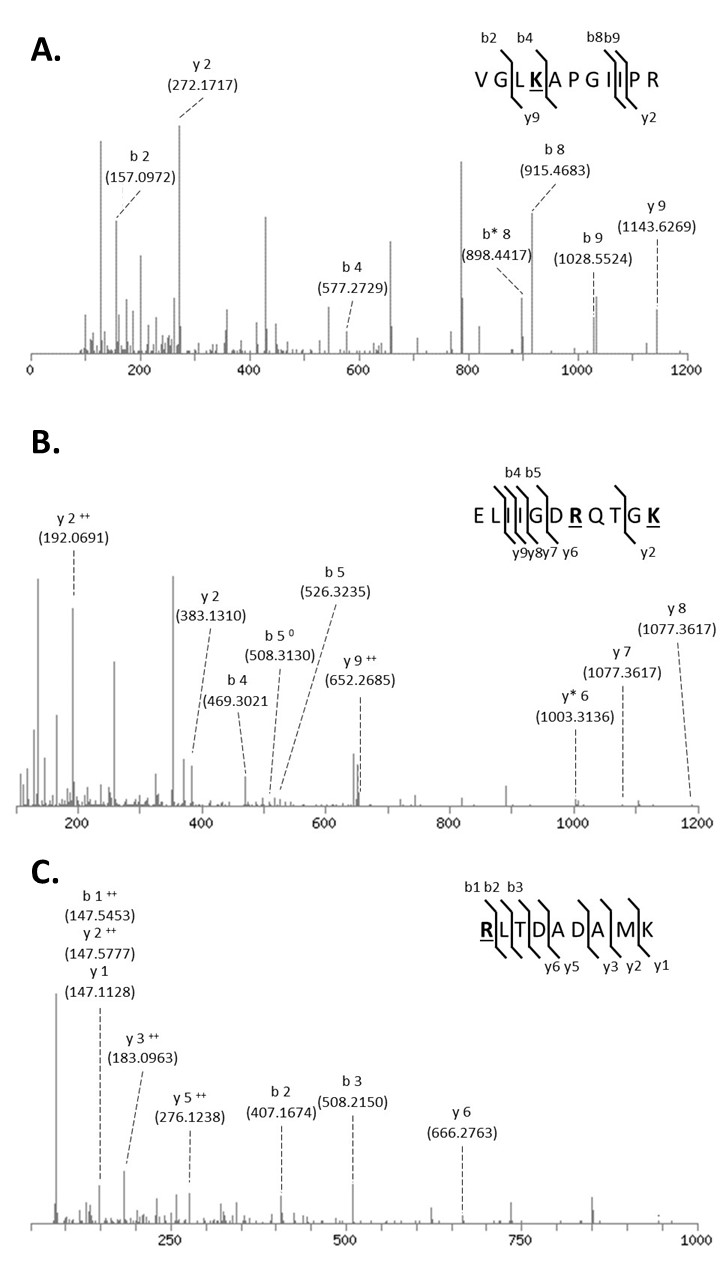

Supplement: Supplementary file 4 — Figure S3. MS/MS spectrum of ATP5A peptides showing carbonylation on K132 (A), R171 and K175 (B), and R219 (C) residue. Underlined alphabets refer to residues with carbonylation. (JPG 97 kb) [file 12953_2019_149_MOESM4_ESM.jpg]
