# Supplementary material for: Hepatic protein Carbonylation profiles induced by lipid accumulation and oxidative stress for investigating cellular response to non-alcoholic fatty liver disease in vitro
Source: Proteome Sci. 2019 Mar 27;17:1. doi: 10.1186/s12953-019-0149-9 (PMC6438040; doi:10.1186/s12953-019-0149-9)
Supplement: Supplementary file 3 — Table S1. Carbonylated sites identified by mass spectrometry of carbonylated proteins detected with redox proteomics. (DOCX 15 kb) [file 12953_2019_149_MOESM3_ESM.docx]

Table S1 Carbonylated sites identified by mass spectrometry of carbonylated proteins detected with redox proteomics

| Protein name | Identified peptides | Carbonylated sites |
| --- | --- | --- |
| L-lactate dehydrogenase A | GEMMDLQHGSLFLRTPK | R-73, K-76 |
|  | LGVHPLSCHGWVLGEHGDSSVPVWSGMNVAGVSLKTLHPDLGTDK | P-199, P216 |
|  | KSADTLWGIQK | T-322 |
| ATP synthase subunit alpha | VGLKAPGIIPR | K-132 |
|  | ELIIGDRQTGK | R-171, K-175 |
|  | RLTDADAMK | R-219 |
| Proteosome activator complex subunit 2 | AKPCGVRLSGEAR | R-8, R-14 |
|  | APLDIPIPDPPPKDDEMETDK | P-63, K-82 |
| Transketolase | AVELAANTKGICFIR | T-464, R-471 |
| Tubulin beta chain | MAVTFIGNSTAIQELFKR | T-366 |
| Serpin B6 | NVFFSPMSMSCALAMVYMGAKGNTAAQMAQILSFNK | T-49 |
